# AI-Led Mental Health Support (Wysa) for Health Care Workers During COVID-19: Service Evaluation

**DOI:** 10.2196/51858

**Published:** 2024-04-19

**Authors:** Christel Lynne Chang, Chaitali Sinha, Madhavi Roy, John Chee Meng Wong

**Affiliations:** 1 Yeo Boon Khim Mind Science Centre National University of Singapore Singapore Singapore; 2 Department of Psychological Medicine Yong Loo Lin School of Medicine National University of Singapore Singapore Singapore; 3 Wysa Inc Boston, MA United States

**Keywords:** AI, app, application, artificial intelligence, COVID-19, digital, health care workers, mental health, pandemic, Wysa

## Abstract

**Background:**

The impact that the COVID-19 pandemic has had on health care workers’ mental health, in particular, cannot be ignored. Not only did the pandemic exacerbate mental health challenges through elevated stress, anxiety, risk of infection, and social isolation, but regulations to minimize infection additionally hindered the conduct of traditional in-person mental health care.

**Objective:**

This study explores the feasibility of using Wysa, an artificial intelligence–led mental health app, among health care workers.

**Methods:**

A national tertiary health care cluster in Singapore piloted the use of Wysa among its own health care workers to support the management of their mental well-being during the pandemic (July 2020-June 2022). The adoption of this digital mental health intervention circumvented the limitations of in-person contact and enabled large-scale access to evidence-based care. Rates and patterns of user engagement were evaluated.

**Results:**

Overall, the opportunity to use Wysa was well-received. Out of the 527 staff who were onboarded in the app, 80.1% (422/527) completed a minimum of 2 sessions. On average, users completed 10.9 sessions over 3.80 weeks. The interventions most used were for sleep and anxiety, with a strong repeat-use rate. In this sample, 46.2% (73/158) of health care workers reported symptoms of anxiety (Generalized Anxiety Disorder Assessment-7 [GAD-7]), and 15.2% (24/158) were likely to have symptoms of depression (Patient Health Questionnaire-2 [PHQ-2]).

**Conclusions:**

Based on the present findings, Wysa appears to strongly engage those with none to moderate symptoms of anxiety. This evaluation demonstrates the viability of implementing Wysa as a standard practice among this sample of health care workers, which may support the use of similar digital interventions across other communities.

## Introduction

During the COVID-19 pandemic, health care systems and workers were under immense pressure as the rates of infection surged. Health care workers experienced elevated levels of stress and psychological burden [1], due to factors such as excessive work hours and an increased risk of infection [2,3]. In a study of Wuhan’s frontline health care workers’ mental health during this pandemic, respondents reported an increase in symptoms of depression and anxiety, and 59% reported moderate to severe perceived stress [4]. Chew et al [5] examined the mental health of health care workers in Singapore and India and found a lower prevalence of psychological symptoms than reported elsewhere but a high prevalence of physical symptoms. These physical symptoms could reflect somatization, as psychological outcomes and the presence of physical symptoms were significantly associated. In a separate study, Tan et al [6] reported that nonmedical health care workers such as allied health professionals, technicians, or administrators had a higher prevalence of anxiety compared to medical staff such as physicians and nurses. This highlights the importance of attending to the mental health of not just frontline medical workers but health care workers as a whole.

Meanwhile, efforts to reduce the spread of COVID-19 have led to nationwide and global lockdowns, resulting in the digitalization of many services. Mental health practitioners likewise adopted web-based strategies to continue supporting clients. Yet, in a time with many in isolation and a worldwide escalation in mental health concerns [7], the urgency for the mainstream adoption of scalable digital mental health services has intensified. In addition to serving as an ad hoc solution during this pandemic, digital mental health interventions through web-based or mobile platforms may provide unique benefits beyond traditional services such as in-person counseling or therapy [8]. For instance, they improve the accessibility and cost of mental health services since traditional mental health interventions are typically subject to standard clinic hours and may not be affordably priced. With digital interventions, individuals can receive on-demand support in moments of crisis, beyond the boundaries of the clinic or therapy room. Digital services can additionally circumvent the stigma-related barriers to mental health treatment since they can be accessed discreetly and at one’s own convenience. In these ways, digital interventions can serve as complements to traditional mental health services, provide interim services for those waiting to receive in-person treatment, and provide new services for those facing barriers to accessing existing clinical interventions [9,10]. Mental health care systems could thus benefit from incorporating digital interventions as part of standard care practices. As an acknowledgment of these advantages, a national tertiary health care division in Singapore piloted the use of Wysa, which is an artificial intelligence (AI)–enabled mental health app among their own health care staff during the COVID-19 pandemic.

Wysa is a mental health mobile app, accessible on the Apple App Store and Google Play Store, designed to address various mental health concerns through self-guided interventions. The app features rule-based AI through a conversational chatbot. This facilitates user interactions, offering personalized support and interventions by comprehending user responses and directing them to relevant resources. Wysa’s AI models operate within a rule-based framework, ensuring clinical safety and adherence to predefined scripts. There is also an option to connect with a human coach, which supplements the app’s fully automated functions.

Due to the primarily self-guided nature of the app, the interventions it provides can be administered to large populations at minimal cost. Moreover, Linardon et al [11] found in their meta-analysis that self-guided interventions significantly surpassed waitlist and active controls in efficacy for reducing symptoms of depression and anxiety. Previous studies examining the utility of mental health mobile apps found Wysa to have high usability and engagement ratings [12,13]. In previous studies, general populations and patients [14] with symptoms of anxiety and depression reported improvements with the use of Wysa. This study serves to evaluate the experience of using Wysa among a population of health care workers during the pandemic.

Wysa is DCB.0129 compliant (the National Health Services [NHS] Clinical Risk Management Standards under the Health and Social Care Act 2012), and its information security management system (ISMS) and privacy information management system (PIMS) have been audited and certified by the British Standards Institution (BSI) for ISO 27001 and ISO 27701. The parent Wysa Android and iOS apps are certified as CE (European Conformity) Medicines and Healthcare Products Regulatory Agency (MHRA) Class 1 Medical Devices. Additionally, Wysa ensures that all user data are encrypted at rest in the storage servers and are hosted by industry leaders who have stringent General Data Protection Regulation (GDPR) and HIPAA (Health Insurance Portability and Accountability Act)–compliant security programs and controls.

## Methods

### Overview

In this pilot exercise, health care staff from a national tertiary health care cluster were provided access to Wysa through paid licenses. Data regarding this community’s use of Wysa and feedback were obtained over a period of 2 years, from July 2020 to June 2022. Advertisements were internally disseminated to the institutions’ staff to encourage them to activate their licenses and engage with the app to support their mental well-being.

### Wysa App

While users interacted with the Wysa chatbot, Wysa would prompt them to indicate which challenges they would like support with. The life challenges disclosed by the present sample of health care workers are given in Table 1. These challenges were predetermined categories that users were able to select, and they present an overview of the key concerns that this population sought help with through the chatbot.

Table 2 displays various categories of emotions reported by users during organic interactions with the chatbot. For interpretative purposes, the specific emotions mentioned in users’ chat messages were identified and combined into categories based on similarity. This enables insight into the range of emotions experienced by these users, which yields granularity to better understand the state of mind they were in as they engaged with the app.

**Table 1:** Health care workers self-reported life challenges while using the Wysa Digital App during the COVID-19 pandemic over 2 years.

User-reported life challenge	Unique users, n (%)
Energy and sleep	531 (97.2)
Self-esteem	404 (73.9)
Relationships	317 (58)
Stress and anxiety	307 (56.1)
Life events	118 (21.6)
Low mood and depression	125 (22.9)
Trauma and loss	71 (13)

**Table 2:** Emotions self-disclosed by health care workers during Wysa chatbot interaction over 2 years during the COVID-19 pandemic.

Emotion valence and emotion category		Count, n
**Negative valence emotions**		
	Low mood and loneliness	368
	Stress and worry	321
	Anger	215
	Fatigue and pain	150
**Positive valence emotions**		
	Happiness	466
	Confidence	222
	Improved mood	177
	Excitement	88
	Gratitude	39

### Ethical Considerations

The conduct of this service evaluation was granted institutional review board exemption by the National University Health System, and the institutional review board’s requirement for informed consent was waived for this posthoc descriptive analysis. All user data analyzed as part of this service evaluation was anonymous, and user data remained fully confidential. The users who engaged with the app were not provided with additional compensation, as the app use was provided to them for free to engage with as they pleased. Users were not systematically recruited to engage with the app, which enabled an evaluation of genuine receptiveness to the app and engagement rate.

## Results

### Digital App Engagement

Out of the 500 licenses purchased by the health care institution, 527 users were onboarded to the app. The discrepancy in numbers is due to the Wysa system disregarding users who had logged in only briefly without further engagement. Of the 527 health care staff who were onboarded, 495 users completed at least 1 full session, and 422 completed 2 or more sessions. One session is defined as a full interaction with the Wysa chatbot or an intervention tool. This indicates a high engagement rate of 93.9% engaging at least once and 80.1% engaging at least twice beyond onboarding. On average, the number of in-app sessions completed by each of the 495 active app users over this period of 2 years was 10.9 (SD 23.3), and the number of weeks of use was 3.80 (SD 5.58). The average number of messages sent by a user was 66.3 (SD 147) and the average number of messages exchanged in each session was 6.25 (SD 8.10). The weeks of use were not necessarily consecutive, although 40.5% of users used Wysa consecutively for at least 3 weeks.

From October 28-30, 2020, a “gratitude challenge” was run in which daily emails prompted users to show gratitude to important people in their lives. Wysa also offered shareable gratitude cards for users to share with their loved ones. This “challenge” was designed to facilitate a boost in engagement with mental health support. During this period, 53 users engaged with the app for an average of 2.30 (SD 1.60) sessions. In the 3 days before the challenge period (October 25-27), there were 31 users who used the app for 2.03 (SD 1.69) sessions on average. Out of these users, 16 of them returned during the period of the challenge. In the 3 days after the challenge period (October 31-November 2), 44 users used the app (mean 2.26, SD 2.38). Of these 44 users, 25 of them were returning users who had engaged during the challenge period. This data may suggest that the challenge was successful in bolstering user engagement, since there was a 71% increase in engagement during the challenge compared to 3 days before, and the number of users who engaged with Wysa after the challenge period had increased by 41.9% compared to before. Initiatives similar to this “challenge” implemented recurrently may have the potential to promote sustained engagement.

While engaging in sessions, users were prompted to provide feedback on their present interaction and experience on a scale of 1-5. Feedback was given by 99 users on 234 sessions at a response rate of 20% (mean 4.07, SD 0.95). Of these, 218 sessions received scores of 3 and above, while 213 sessions received scores of 4 and above, suggesting a strong positive feedback rate of 93.2% and 91% receiving highly positive feedback, respectively. Of the 495 active app users, 158 individuals responded to the Generalized Anxiety Disorder Assessment-7 (GAD-7) and the Patient Health Questionnaire-2 (PHQ-2) screening questionnaires within the app. User-experience feedback was given by 59 out of the 158 users. This response rate of 37.3% is higher than the overall sample’s rate of 20%. The user-experience feedback given by these users as a function of their GAD-7 and PHQ-2 scores is shown in Table S1 in Multimedia Appendix 1. Approximately 30%-40% of individuals falling into each severity category gave feedback, with no obvious differences in the rate of response between categories.

### Clinical Utility

**Figure 1:**
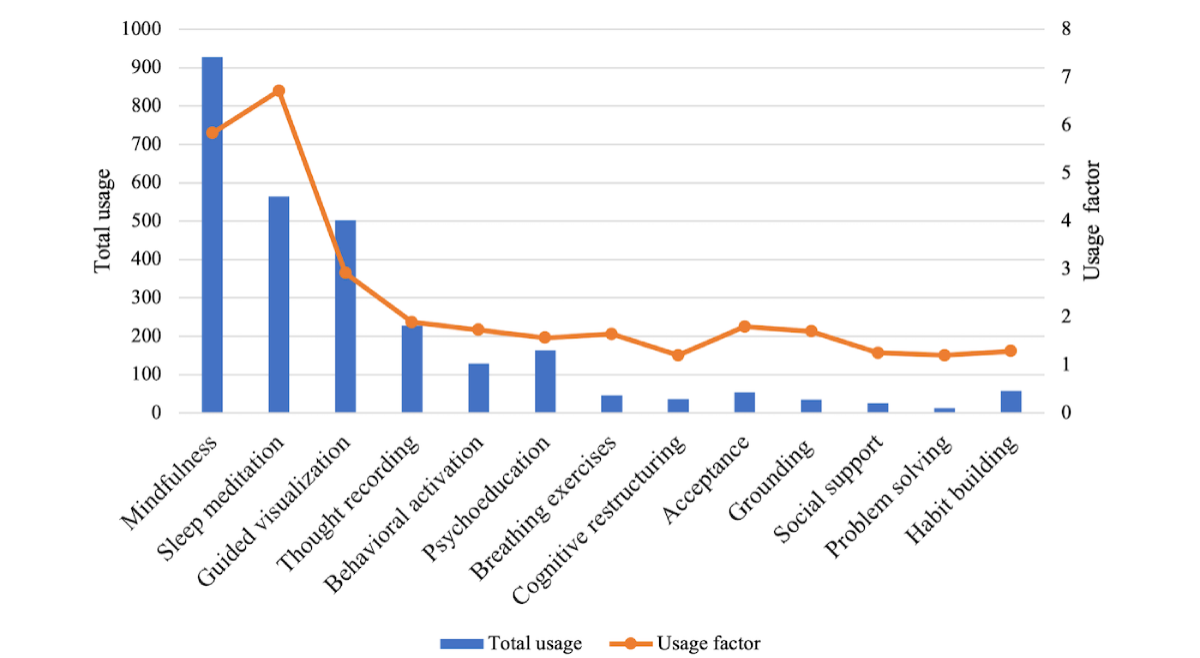
Pattern of Wysa intervention use by health care workers over 2 years during the COVID-19 pandemic.

Wysa contains an assortment of self-guided interventions, which each have a collection of interventions categorized by relevance to a circumstance or emotion. The interventions consist of AI-enabled conversations that target specific interventions. Table 3 gives a breakdown of how the Wysa interventions were used among this community of health care workers. Descriptions of the various interventions can be found in Multimedia Appendix 2. The data are based on users who actively sought help from these interventions by directly clicking on them. The number of unique users provides an indication of use pattern, that is, how many distinct individuals contributed to the total use compared to repeat users. Figure 1 illustrates this ratio (use factor) of unique users to total use. The higher the use factor, the higher the rate of repeat use. For instance, the number of unique users for sleep meditation interventions was 84, suggesting that this handful of users repeatedly engaged with this intervention a total of 564 times, with a high use factor of 6.7. In comparison, although guided visualization interventions were used a total of 502 times (an overall use comparable to that of sleep meditations), these interventions were used repeatedly by a larger number of 172 users with a use factor of 2.9. As such, it appears that a smaller subset of individuals repeatedly used interventions for sleep meditation, whereas guided visualizations were used by a wider range of individuals with a lower use factor.

Unsurprisingly, since sleep difficulties tend to co-occur alongside distressing events, interventions for sleep were overwhelmingly sought after, with high total use levels (Table 3). The next most popular interventions were for anxiety, especially health anxiety, stress, and self-esteem. These use patterns generally coincide with the top life challenges, as reported in Table 1. Furthermore, the popularity of the health anxiety intervention aligns with the backdrop of the COVID-19 pandemic.

GAD-7 and PHQ-2 screening tools were embedded within the app to screen for anxiety and depressive symptoms, respectively, which users were prompted to respond to after their very first chatbot conversation. This initial screening helped to capture baseline indications of the present population. Of the 495 active app users, 158 individuals responded to these questionnaires at this time point (158/495, 31.9% response rate). Table S1 in Multimedia Appendix 1 presents a breakdown of the prevalence of anxiety and depressive symptoms in this sample according to severity (number of users). Overall, 46.2% (73/158) reported symptoms of anxiety, and 15.2% (24/158) were likely to have symptoms of depression.

**Table 3:** Use of Wysa interventions among health care workers over 2 years during the COVID-19 pandemic.

Interventions	Total use, n	Unique users, n
Mindfulness	928	159
Sleep meditation	564	84
Guided visualization	502	172
Thought recording	227	120
Behavioral activation	128	74
Psychoeducation	163	104
Breathing exercises	46	28
Cognitive restructuring	36	30
Acceptance	54	30
Grounding	34	20
Social support	25	20
Problem solving	12	10
Habit building	58	45

The average use rates (number of weeks and sessions) according to users’ GAD-7 and PHQ-2 scores are also displayed in Table S1 in Multimedia Appendix 1. The average number of weeks that users engaged with Wysa provides an indication of how long their symptoms or interest in using Wysa lasted, while the average number of sessions gives an estimate of use frequency. To illustrate, this data demonstrates that health care workers in this sample with moderate anxiety engaged with Wysa for an average of 6.45 weeks. Over these 6.45 weeks, users completed an average of 20.2 sessions.

Individuals with no, mild, or moderate anxiety used Wysa for a high mean number of sessions (mean_total_ 19.5) and weeks of use (mean_total_ 6.04), almost 2 times higher than the overall active app user average of 10.9 and 3.80, respectively, while there was a limited sample of individuals with symptoms of severe anxiety. Users who were likely to have symptoms of depression engaged with Wysa more and for a marginally longer duration on average than those who were unlikely, as seen in Table S1 in Multimedia Appendix 1. This finding is promising, as it suggests that Wysa may be more beneficial and motivating to use for individuals with symptoms of depression.

Regardless of GAD-7 or PHQ-2 severity, the most popular interventions used were for sleep, especially for deep sleep, and anxiety, especially for health anxiety (Table S1 in Multimedia Appendix 1). Users falling into the moderate and severe GAD-7 categories were more targeted in using Wysa, specifically to help with sleep and anxiety, and did not explore the other interventions as much as other users. On the other hand, users with no or mild anxiety explored more of Wysa’s other interventions. Likewise, users who were unlikely to have symptoms of depression engaged with a wider variety of interventions than those who were screened as likely. In general, this data emphasizes the popularity of sleep and anxiety interventions among this community and implies that greater severity of mental health symptoms is associated with more targeted engagement with the app.

## Discussion

### Overview

Due to heightened restrictions on in-person contact, the COVID-19 pandemic has brought to the forefront the current limitations of mental health care delivery. As such, the advent of the pandemic has proved a catalyst for new digital initiatives to be facilitated [15]. The present community case study is a service evaluation of a pilot exercise within a national tertiary health care division in Singapore using a self-guided, AI-enabled mental health app (Wysa) during the COVID-19 pandemic. The availability of Wysa for this population of health care staff to use was well-received, particularly against this backdrop of pandemic-related anxiety. Even beyond the height of the pandemic (2020-21), the continued use of Wysa by this community suggests that this digital mental health solution has merits surpassing those of temporary pandemic-specific support. The high initial engagement rate of 93.9% of onboarded users completing at least 1 in-app session may have been due to this intervention being introduced through a trusted source—a health care institution or employer [14]. Nevertheless, 80.1% of users returned to engage with the app voluntarily for a full second time, and the mean number of in-app sessions completed was 10.9 for an average of 3.80 weeks. In-app use reflects recurring engagement with Wysa, but users could also engage with the chatbot through the website through a widget. As a whole, the data demonstrates that users may have experienced some real or perceived benefit, which motivated their continued engagement with the app.

Among this community, 46.2% reported symptoms of anxiety, and 15.2% were likely to have symptoms of depression. These prevalence rates are comparable to those of other health care communities in other parts of the world during the COVID-19 pandemic. For instance, these rates are similar to the 44.6% that had symptoms of anxiety (GAD-7) and 14.8% that had moderate to severe depression (PHQ-9) in a sample of health care workers from China [16]. However, compared to a population of health care workers in the United Kingdom following the pandemic’s first peak, the present sample’s anxiety prevalence was higher than the UK sample’s 34.3%, but depression prevalence was lower than the UK sample’s 31.2% (PHQ-4) [17]. Following a similar trend, the present sample’s rate of anxiety was slightly higher than the pooled prevalence rate of 40%, but depression prevalence was lower than the pooled prevalence of 37% from a meta-analytic study of health care workers across 19 countries during this pandemic [18]. Compared to the general population during the COVID-19 pandemic, this community of health care workers’ anxiety and depression symptom prevalence rates were similarly higher and lower than the global prevalence rates of around 27.3%-31.9% and 33.7%, respectively [19,20].

In general, the intervention use patterns (Table 3) concur with the user-reported life challenges (Table 1). The data regarding the patterns of intervention use indicates that “for deep sleep” was the most popular intervention used by the greatest number of users repeatedly for the most number of times. “Sleep sounds” was used the second-largest number of times, but only by a handful of unique individuals. This suggests that an intervention for sleep sounds may not be attractive to a wide range of users, yet for those who decide to try it, it appears motivating to use, thus resulting in its high repeat-use rate. The “health anxiety” intervention was the next most popular intervention, used by almost as many unique users who engaged with the intervention “for deep sleep.” The popularity of this intervention may be idiosyncratic to the COVID-19 pandemic period. Overall, this data implies that this community of health care workers primarily sought help from Wysa for sleep concerns and anxiety or found these interventions to be the most beneficial. The analysis of most used interventions according to GAD-7 and PHQ-2 severity further indicates that this is true regardless of their mental health status.

Users in this community with moderate to severe symptoms of anxiety were more targeted in using Wysa specifically to help with sleep and anxiety and did not explore the other interventions as much as other users. This was reasonable to expect, as individuals with anxiety often have co-occurring sleep difficulties and would likely allot more attention to addressing these key concerns. Users with no or mild anxiety explored more of Wysa’s other interventions. Their engagement in a greater range of interventions could explain why these individuals continued to use Wysa despite their lack of or few anxiety symptoms. Likewise, those who were unlikely to have symptoms of depression engaged with a wider variety of interventions than those who were likely. Although this may partially be due to a large number of individuals falling under the unlikely category, thus contributing to the heterogeneity of use patterns, engagement in a large range of interventions by users with fewer mental health concerns may reflect their use of this app in a manner relating to principles of positive psychology [21] to further enhance one’s quality of life. This contrasts with the ostensibly treatment-targeted approach taken by users with greater psychopathological challenges. The dichotomous manner of use noted here may attest to the breadth of what the Wysa app could support users with.

Individuals with mild or moderate anxiety symptoms may sufficiently benefit from using Wysa in a self-directed manner, thus leading to high use and positive feedback rates. This hypothesis may be supported by Karyotaki and colleagues’ review [22], which found that individuals with more severe depression may benefit more from guided interventions involving human support, whereas individuals with mild or subthreshold symptoms may be adequately supported by self-guided interventions. Although, in the present sample, this does not hold true with regard to depressive symptoms, since users likely to have depression engaged with Wysa at comparable rates to those who were unlikely (Table S1 in Multimedia Appendix 1). This is an encouraging finding, as it suggests that individuals with symptoms of depression may have derived benefit from, or at least enjoyed, continuing to use this app to support their well-being. Yet, the depression severity in this study was determined using the PHQ-2. More accurate and potentially different results might have been obtained if the PHQ-9 had been used. In addition, individuals with no symptoms of anxiety as reported on the GAD-7 engaged with Wysa at high rates, on par with users with mild to moderate symptoms. This may indicate that Wysa is motivating to use for individuals that may not have any overt mental health struggles, which could imply that Wysa is able to support users in maintaining their positive mental health or to flourish further in eudaimonia, as proposed in a previous paragraph. Nonetheless, grounded conclusions could only be determined through direct qualitative inquiry or more rigorous quantitative pre-post analyses, which this evaluation was not able to perform due to methodological constraints. To summarize, Wysa appears to be beneficial or motivating to use for individuals with no, mild, or moderate anxiety, whereas individuals with severe anxiety symptoms may need to seek other forms of guided clinical care.

As there was a 71.0% increase in use of the app during the “gratitude challenge” period, the challenge appeared to be successful in boosting app engagement. Extant literature provides support for the advantages of practicing gratitude, from experimental research that found gratitude to enhance the psychological benefits of social support [23] to a randomized controlled trial of a gratitude intervention in which gratitude effectively improved well-being [24]. A review by Bono and Sender [25] suggests that gratitude’s ability to amplify social benefits and motivate self-improvement makes it foundational to human development. This powerful potential may have propelled the increase in app engagement. Future endeavors to implement similar digital mental health interventions may benefit from a greater volume of similar initiatives at regular intervals. However, to assess the efficacy of such initiatives more accurately, more robust research is needed to isolate the effect of the challenge from other confounding variables. In addition, the present sample of users who responded to the GAD-7 and PHQ-2 screening questionnaires was too small to draw any conclusions regarding the mental health status of individuals that these types of “challenges” may work best on. Future studies should evaluate this further.

Altogether, the present service evaluation shows promising evidence to endorse the implementation of digital mental health interventions as standard practice, as the high repeat-use rates for Wysa’s interventions corroborate with the strong positive feedback and user engagement rates. Health care systems could consider implementing similar digital interventions as part of routine care for patients or to support their own staff internally. Clinicians may leverage these interventions to improve the capacity of mental health care service delivery, since these digital interventions have the potential to greatly increase access to evidence-based psychological support. For such ventures to be implemented, a systematic approach could be taken by first determining the appropriate digital solution for the community in question [9]. Using an integrated tool such as Wysa with its diverse assortment of interventions would be able to cover a wide variety of needs, possibly ranging from treatment-targeting solutions to the eudaimonic enhancement of well-being. Yet, as seen in this study, the majority of this community’s users were chiefly interested in the interventions for sleep and anxiety, regardless of the severity of their symptoms. If the community has more specific needs, perhaps more targeted apps or other digital interventions could be considered.

### Limitations

The evaluation of the pilot exercise outlined in this study has several limitations. First, intervention use data from this population was based on users directly clicking on the interventions. As such, this was not able to capture the interventions used that were suggested by Wysa through chatbot conversations. Next, the PHQ-2 was used to collect data on users’ depressive symptoms. However, the PHQ-9 may have been able to yield more precise information. In addition, comparison between baseline and subsequent scores would be required to generate more reliable quantitative results that elucidate Wysa’s ability to moderate symptoms of poor mental health. In the present exercise, insufficient users responded at follow-up screenings in order to conduct meaningful pre-post analyses. Moreover, there was only a moderate response rate of 31.9% to the screening questionnaires at baseline. Conclusions drawn from the present data may thus not be representative of the entire community that participated, and the small sample size of respondents is a limitation in itself. The users who responded to the screening interventions also appeared to contribute to a large proportion of the overall population’s engagement with Wysa, since this sample’s average use rates were approximately twice as high as the overall population’s average rates. This may further indicate a biased sample. Furthermore, although the present data demonstrates the propitiousness of the “gratitude challenge,” its true efficacy in boosting engagement could not be ascertained since a controlled experimental design would be necessary.

Another limitation is that assumptions regarding reasons for engagement with Wysa are speculative; for example, in assuming that users who repeatedly used the app benefitted from its use or that the number of weeks of use reflects how long their symptoms lasted. However, it is possible that these users might not have seen much benefit, yet they were patient in attempting to engage with Wysa. This would thus be more telling of the user’s character or personality than of the app’s effectiveness. Similarly, it is not possible to determine whether the ubiquitous popularity of the sleep and anxiety interventions was due to users being mainly concerned with these challenges or whether this phenomenon was a result of users’ expectations regarding what an app could plausibly help with. To yield more reliable conclusions, qualitative data would be required, collected through feedback through the app or a follow-up focus group discussion. Conducting a focus group may further enable the generation of insight into the lived experiences of these users. This may be especially useful to illuminate the experience of those with severe anxiety symptoms as they attempted using Wysa, to deduce the actual reasons for their low rates of engagement, and for those with no anxiety, to discern whether Wysa simply sustained their mental health or was able to promote even greater degrees of well-being. Nevertheless, due to the anonymous nature of the data, reidentification of the users to follow up with would not be possible. For more rigorous research, a dedicated study would have to be designed with ethical approval and consent obtained from health care workers to participate. Future studies could thus be developed based on the promising findings from this community service evaluation.

## Appendix

Multimedia Appendix 1 Table S1. Health care workers’ anxiety and depressive symptom prevalence and associated Wysa app use rates, feedback, and interventions used over 2 years during the COVID-19 pandemic.

Multimedia Appendix 2 Description of Wysa interventions.

## Abbreviations

AI: artificial intelligence
BSI: British Standards Institution
CE: European Conformity
GAD-7: Generalized Anxiety Disorder Assessment-7
GDPR: General Data Protection Regulation
HIPAA: Health Insurance Portability and Accountability Act
ISMS: information security management system
MHRA: Medicines and Healthcare Products Regulatory Agency
NHS: National Health Services
PHQ-2: Patient Health Questionnaire-2
PIMS: privacy information management system
