# Supplementary figures and images for: AI-Led Mental Health Support (Wysa) for Health Care Workers During COVID-19: Service Evaluation
Source: JMIR Form Res. 2024 Apr 19;8:e51858. doi: 10.2196/51858 (PMC11034576; doi:10.2196/51858)

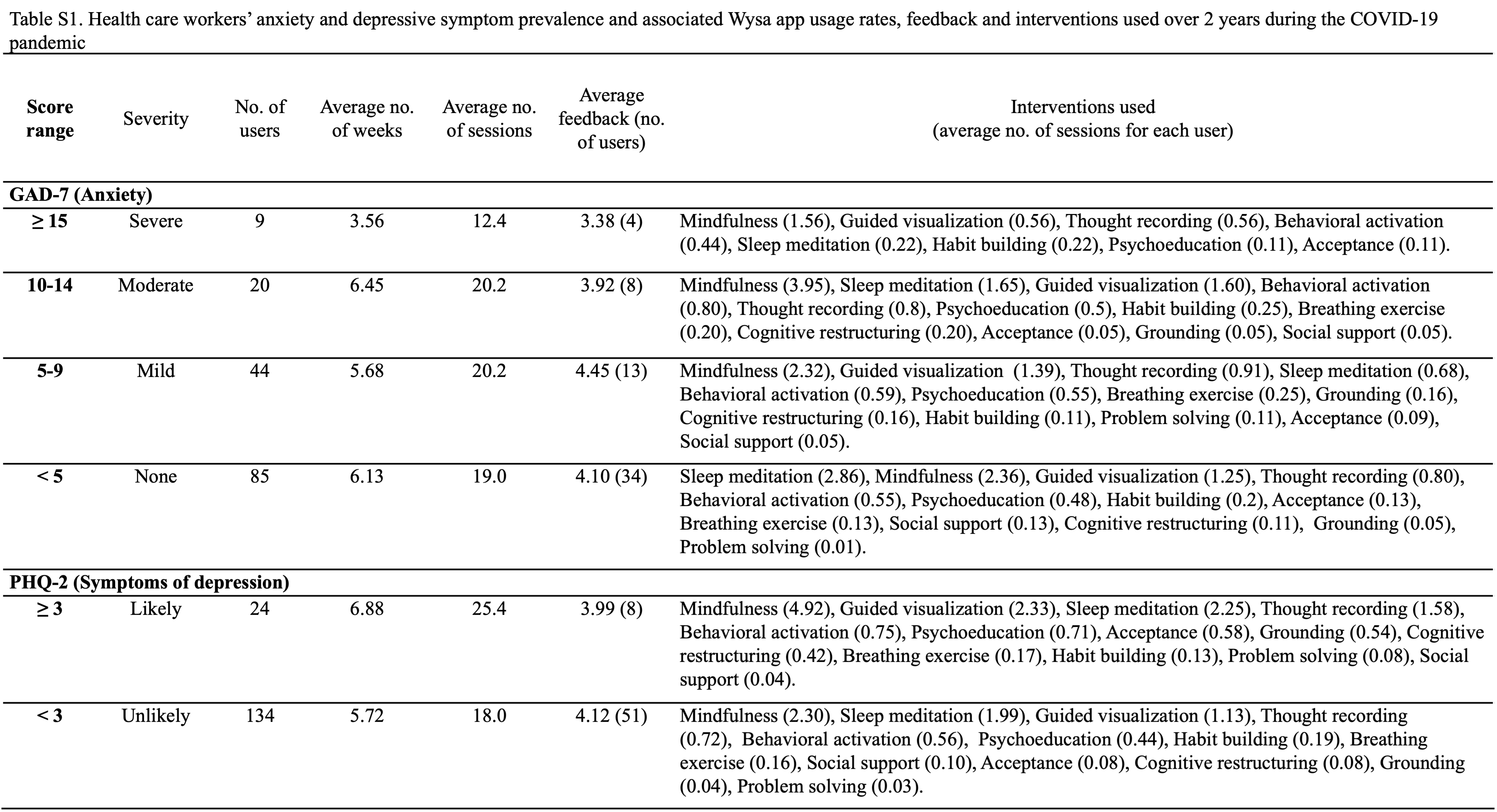

Supplement: Multimedia Appendix 1 [file formative_v8i1e51858_app1.png]
