# Supplementary material for: AI-Led Mental Health Support (Wysa) for Health Care Workers During COVID-19: Service Evaluation
Source: JMIR Form Res. 2024 Apr 19;8:e51858. doi: 10.2196/51858 (PMC11034576; doi:10.2196/51858)
Supplement: Multimedia Appendix 2 [file formative_v8i1e51858_app2.docx]

## Names of interventions and corresponding descriptions

### Mindfulness

These interventions aim to develop and enhance self-compassion, self-control, objectivity, affect tolerance, equanimity, mindful detachment, inner strength, and the ability to be strong in the face of chaos, change, or uncertainty. This is achieved through exercises focusing on meditation, body scans, and thought observation.

### Sleep Meditation

These interventions help users sleep deeply through relaxing meditations, sleep stories, and sleep sounds/audios.

### Guided Visualization

These interventions include exercises such as guided meditations and breathing exercises, in order to help users find relief by visualizing, thinking through their worries, and looking at situations through a new lens.

### Thought Recording

Thought recording is an intervention that helps users pay attention to their thoughts, recognize their unhelpful thoughts, and work towards changing them.

### Behavioral Activation

These interventions aim to increase engagement in activities that help users reach a more positive emotional state, and help users experience pleasure or mastery.

### Psychoeducation

These tools provide information about emotional states, motivation, stress, and mental health concerns to users.

### Breathing Exercises

These include deep breathing exercises and diaphragmatic breathing exercises to increase energy levels, reduce anxiety, calm the mind, and relieve stress.

### Cognitive Restructuring

Cognitive restructuring helps users think through their worries, guides them to reframe their thoughts in a helpful way, and helps them become more accepting of their situation.

### Acceptance

Exercises focusing on acceptance helps the user cultivate the strength and awareness to accept negative emotions with mental resilience.

### Grounding

Grounding is a mindfulness-based technique that helps users manage panic, and puts their minds at ease by helping them focus on the here and now and increasing their awareness.

### Social Support

During tough times, a social support system - friends, family, peers, or some useful activities can support us practically and emotionally and even help reduce depression and anxiety. These interventions help users understand the thoughts that make them feel alone and guide them through overcoming them.

### Problem-Solving

These interventions help users define their problems, analyze them objectively, and manage their emotions to work towards a solution.

### Habit Building

These interventions help users build habits to improve their daily routines and enhance feelings of gratitude and capacity for resilience
